# CXCR7 Antagonism Reduces Acute Lung Injury Pathogenesis

**DOI:** 10.3389/fphar.2021.748740

**Published:** 2021-11-05

**Authors:** Laetitia Pouzol, Anna Sassi, Nadège Baumlin, Mélanie Tunis, Daniel S. Strasser, François Lehembre, Marianne M. Martinic

**Affiliations:** Idorsia Pharmaceuticals Ltd., Allschwil, Switzerland

**Keywords:** acute lung injury, acute respiratory distress syndrome, immunomodulation, CXCR7, CXCR3, CXCR4, CXCL11, CXCL12

## Abstract

Loss of control in the trafficking of immune cells to the inflamed lung tissue contributes to the pathogenesis of life-threatening acute lung injury (ALI) and its more severe form, acute respiratory distress syndrome (ARDS). Targeting CXCR7 has been proposed as a potential therapeutic approach to reduce pulmonary inflammation; however, its role and its crosstalk with the two chemokine receptors CXCR3 and CXCR4 via their shared ligands CXCL11 and CXCL12 is not yet completely understood. The present paper aimed to characterize the pathological role of the CXCR3/CXCR4/CXCR7 axis in a murine model of ALI. Lipopolysaccharide (LPS) inhalation in mice resulted in the development of key pathologic features of ALI/ARDS, including breathing dysfunctions, alteration in the alveolar capillary barrier, and lung inflammation. LPS inhalation induced immune cell infiltration into the bronchoalveolar space, including CXCR3^+^ and CXCR4^+^ cells, and enhanced the expression of the ligands of these two chemokine receptors. The first-in-class CXCR7 antagonist, ACT-1004-1239, increased levels of CXCL11 and CXCL12 in the plasma without affecting their levels in inflamed lung tissue, and consequently reduced CXCR3^+^ and CXCR4^+^ immune cell infiltrates into the bronchoalveolar space. In the early phase of lung inflammation, characterized by a massive influx of neutrophils, treatment with ACT-1004-1239 significantly reduced the LPS-induced breathing pattern alteration. Both preventive and therapeutic treatment with ACT-1004-1239 reduced lung vascular permeability and decreased inflammatory cell infiltrates. In conclusion, these results demonstrate a key pathological role of CXCR7 in ALI/ARDS and highlight the clinical potential of ACT-1004-1239 in ALI/ARDS pathogenesis.

## Introduction

Acute lung injury (ALI) and its more severe form, acute respiratory distress syndrome (ARDS), are life-threatening lung diseases that can be the result of different indirect or direct insults to the lung, such as sepsis, trauma, gastric acid aspiration, and pneumonia, including viral pneumonia, such as SARS-CoV-2-induced pneumonia (Wheeler and Bernard, 2007; Gibson et al., 2020). To date, the pathogenesis of these diseases is still not completely understood, and there is no disease-modifying therapy to reduce the high mortality incidence of ARDS (Bellani et al., 2016).

ALI and ARDS are characterized by increased lung vascular permeability, pulmonary edema, diffuse alveolar damage, and recruitment of inflammatory cells to the lungs resulting in clinical symptoms such as hypoxemia, dyspnea, and even severe acute respiratory failure (Ware and Matthay, 2000).

Many chemokines and their receptors, which are key mediators of immune cell trafficking, play a critical role in ALI pathogenesis and in its resolution (Bhatia et al., 2012; Tomankova et al., 2015). Following lung injury, chemokine gradients are established and tightly regulated via complex mechanisms to recruit inflammatory cells to the site of inflammation (Puneet et al., 2005).

CXCR3/CXCR4/CXCR7 and their ligands are overexpressed and heavily implicated in the pathology of a number of inflammatory diseases including pulmonary diseases (Saetta et al., 2002; Petty et al., 2007; Hartl et al., 2008; Costello et al., 2012; Ichikawa et al., 2013; Ngamsri et al., 2017; Liao et al., 2020).

CXCR7, also referred to as ACKR3, is an atypical chemokine receptor, which is mainly expressed on endothelial cells (Berahovich et al., 2014). CXCR7 does not couple with G proteins, but binding of its ligands leads to the recruitment of β-arrestin. CXCR7 functions predominantly as a scavenger receptor for its chemokine ligands CXCL11 and CXCL12 (Naumann et al., 2010), which bind to, and activate, the signaling chemokine receptors CXCR3 and CXCR4, respectively. While CXCR7 is not expressed on leukocytes (Berahovich et al., 2010), its scavenging activity in endothelial cells contributes to the establishment and maintenance of CXCL11 and CXCL12 concentration gradients along which CXCR3^+^ and CXCR4^+^ cells can migrate from the blood toward the inflamed tissue (Lewellis and Knaut, 2012; Quinn et al., 2018; Pouzol et al., 2021).

CXCR3 is a cell surface receptor expressed on subsets of adaptive and innate immune cells such as lymphocytes, natural killer (NK) cells, dendritic cells (DCs), and can be activated by three interferon-inducible chemokine ligands: CXCL9, CXCL10, and CXCL11 (Groom and Luster, 2011). CXCR3–ligand interaction results in various cellular functions including cell migration, proliferation, polarization, and tissue retention (Groom and Luster, 2011).

CXCR4 is expressed on various immune cells including lymphoid and myeloid cells, endothelial cells, and hematopoietic stem cells (Lewellis and Knaut, 2012). CXCL12–CXCR4 signaling results in pleiotropic cellular functions including cell migration, adhesion, proliferation, differentiation, and survival (Quinn et al., 2018).

Blockade of CXCR7 is expected to increase the systemic CXCL11 and CXCL12 levels and, therefore, modulate CXCR3 and CXCR4 signaling activities such as leukocyte chemotaxis and tissue retention (Berahovich et al., 2014; Pouzol et al., 2021). In line with this hypothesis, treatment with CCX771, a CXCR7 functional antagonist, which is known to increase plasma CXCL12 levels (Berahovich et al., 2014), led to reduced alveolar inflammation and lung microvascular permeability in a murine model of ALI (Konrad et al., 2017; Ngamsri et al., 2017). However, this murine model was performed in C57BL/6 mice, which do not express CXCL11 (Sierro et al., 2007). As such, the specific role of CXCR7 in ALI and its indirect effect on CXCR3 and CXCR4 via their shared ligands have not yet been evaluated in an appropriate experimental design. In addition, since so far only CCX771, which recruits β-arrestin upon binding to the receptor, has been used in the ALI model (Zabel et al., 2009), it remains unclear whether the observed efficacy is due to its agonistic activity or its functional antagonism induced by receptor desensitization (Menhaji-Klotz et al., 2020).

To elucidate the role of CXCR7 on pulmonary inflammation, an ALI/ARDS experimental model was established through inhalation of nebulized lipopolysaccharide (LPS) in DBA/1 mice, a strain susceptible to LPS-induced ALI (Alm et al., 2010) and known to express both CXCL11 and CXCL12 (Richard-Bildstein et al., 2020). Since the pathological mechanisms of LPS-induced ALI/ARDS can vary between the early and late phases of the inflammatory response (Domscheit et al., 2020), this model was characterized over time for the main features of ALI/ARDS, namely, lung dysfunction, vascular permeability, inflammatory cell recruitment, and CXCR3/CXCR4/CXCR7 chemokine ligands release. Furthermore, the mechanistic and functional roles of CXCR7 were evaluated over time using the selective and first-in-class CXCR7 antagonist ACT-1004-1239, which blocks CXCL11- and CXCL12-induced β-arrestin recruitment (Richard-Bildstein et al., 2020).

## Materials and Methods

### Mice and Treatment Administration

Male DBA/1 mice were purchased from Janvier Laboratories (Le Genest-Saint-Isle, France) and allowed to acclimatize for at least 7 days before use. The gender of mice was chosen based on previous studies showing that ARDS occurs more commonly in males than in females (Lemos-Filho et al., 2013). Mice had free access to food and drinking water *ad libitum* and were group housed in a light-controlled environment.

The CXCR7 antagonist ACT-1004-1239 was synthetized as previously described (Richard-Bildstein et al., 2020). The compound was formulated in 0.5% methylcellulose (Sigma-Aldrich, Schnelldorf, Germany) and 0.5% Tween 80 (Sigma-Aldrich) in water. ACT-1004-1239 and vehicle (0.5% methylcellulose, 0.5% Tween 80 in water) were administered orally (p.o.), twice a day (b.i.d.) at a volume of 5 ml/kg/administration (10 ml/kg/day) at doses and times indicated in the figure legends. The twice-daily oral administration regimen was based on the pharmacokinetic properties of this compound, which has been shown to be a high-clearance drug in rodents (Richard-Bildstein et al., 2020).

### Murine Model of Lipopolysaccharide-Induced Acute Lung Injury

LPS challenge was performed as previously described (de Souza Xavier Costa et al., 2017). Briefly, mice were exposed to nebulized LPS (*Escherichia coli* O111:B4, purified by phenol extraction; Sigma-Aldrich) at 0.8 mg/ml diluted in NaCl 0.9% (B Braun Medical, Sempach, Switzerland) in a plexiglas chamber connected to a nebulizer (System Assistance Medicale, Ledat, France) for 30 min. Control mice inhaled NaCl 0.9% only. Vehicle or ACT-1004-1239 was given p.o., 1 h prior (preventive setting) or 3 h post inhalation (therapeutic setting).

At different time points indicated in the figure legends (5, 24, 48, 72 h) following LPS or NaCl challenge, mice were euthanized with an overdose (150 mg/kg, intraperitoneally) of pentobarbital (Esconarkon, Streuli Pharma SA, Uznach, Switzerland), and samples were collected.

### *In Vivo* Lung Function

Lung function was measured in unrestrained, conscious, and spontaneously breathing mice by whole‐body plethysmography (Emka Technologies, Paris, France) as previously described (Piali et al., 2017). Briefly, each mouse was placed alone in a calibrated plethysmography chamber, and lung function parameters were recorded for 1 h for baseline assessment. Right after the baseline, ACT-1004-1239 or vehicle was given orally 1 h prior to nebulized LPS or NaCl inhalation. Respiration parameters were measured for 6 h just after the challenge in the plethysmograph. Enhanced pause, Penh, [(Te/RT)‐1)*PEF/PIF, where Te is the expiratory time, RT is the relaxation time, PEF is the peak expiratory flow, and PIF is the peak inspiratory flow) was used as an index of alterations in respiration (Hamelmann et al., 1997; Prada-Dacasa et al., 2020). Data were analyzed using the IOX2 software (Emka) and expressed as area under the curve (AUC), recorded for 30 min and averaged at 5-min intervals. The time indicated in the figure refers to the starting time of the analyzed period (e.g., 60 min refers to the AUC calculated for the 60- to 90-min time interval). The mean Penh AUC baseline measurement was set to 100% for each mouse, and calculated Penh AUC data were expressed as percentage of this mean baseline measurement.

### Bronchoalveolar Lavage Collection

BAL fluid was collected at different time points indicated in the figure legends following LPS or NaCl challenge. BAL was performed by injection of a total volume of 2.25 ml of phosphate-buffered saline (PBS, Bioconcept, Allschwil, Switzerland) supplemented with EDTA (0.5 mM, Gibco, Thermo Fisher Scientific, Waltham, MA, United States) through the mouse incised trachea. After centrifugation, BAL supernatant was collected and kept at −20°C until use. BAL cells were analyzed by flow cytometry.

### Flow Cytometry of the Bronchoalveolar Lavage Cells

BAL cells were stained with the following surface fluorochrome-conjugated monoclonal anti-mouse antibodies: APC-Cy7 anti-mouse CD19 (Clone 6D5; BioLegend, San Diego, CA, United States), APC anti-mouse CXCR3 (Clone CXCR3-173; BioLegend), BV510 anti-mouse CD11b (Clone M1/70; BioLegend), BV605 anti-mouse CD4 (Clone GK1.5; BioLegend), FITC anti-mouse B220 (Clone Ra3-6B2; BD), PB anti-mouse CD45 (Clone 30-F11; BioLegend), PECy7 anti-mouse βTCR (Clone H57-597; BioLegend), PE anti-mouse CXCR4 (Clone 2B11; Invitrogen, Thermo Fisher Scientific), BV650 anti-mouse CD8 (Clone 53-6.7; BioLegend), APC-Cy7 anti-mouse Gr-1 (Clone RB6-8C5; BioLegend), AF700 anti-mouse CD3 (Clone 17A2; BioLegend), FITC anti-mouse CD49b (Clone DX5; BioLegend), BV-605 anti-mouse B220 (Clone RA3-6B2; BioLegend), and PECy7 anti-mouse CD11c (Clone N418; BioLegend). Staining was performed on ice, in the dark, during 45 min after preincubation with Fc receptor blocker (CD16/CD32; BD Biosciences). Dead cells were excluded based on their positive staining with propidium iodide (PI; CAS 25535-16-4; Sigma-Aldrich). Samples were acquired on a CytoFLEX Flow cytometer (Beckman Coulter Life Sciences, Nyon, Switzerland), and data were analyzed using the Kaluza analysis software version 2.1 (Beckman Coulter). Cells were first gated in forward scatter versus side scatter, and doublets were excluded based on forward scatter area–height. From the single cells, dead cells were excluded based on their positive staining with PI. Cell subsets were quantified among viable/CD45^+^ cells (PI^−^, CD45^+^ cells): neutrophils (CD11b^+^, Gr-1^high^ cells), monocytes/macrophages (CD11b^+^, Gr-1^low^ cells), alveolar macrophages (CD11b^int^, SSC^high^), B cells (CD11b^−^, Gr-1^−^, CD49^−^, CD3^−^, B220^+^ cells), plasmacytoid dendritic cells (pDCs) (CD11b^−^, Gr-1^int^, B220^+^, CD11c^+^ cells), T cells (CD11b^−^, B220^−^, CD49b^−^, CD3^+^ cells), natural killer (NK) cells (CD11b^−^, CD3^−^, CD49b^+^), and dendritic cells (DCs) (CD11b^−^, B220^−^, CD49b^−^, CD3^−^, CD11c^+^ cells). The CXCR4^+^ and CXCR3^+^ leukocytes were identified based on the fluorescence minus one control for CXCR4 and CXCR3, respectively. The gating strategies are illustrated in Supplementary Figures S1, S3.

### CXCL12 and CXCL11 Measurement

Whole blood was collected in EDTA-coated tubes (BD Microtainer, Becton Dickinson, Franklin Lakes, NJ, United States) and centrifuged to prepare plasma. After blood and BAL collection, mice were transcardially perfused with PBS/EDTA. Lungs were collected as a whole and kept at −20°C until use. Frozen mouse lungs were homogenized (FastPrep, MP Biomedicals, Illkirch, France) in RIPA buffer supplemented with 1% protease inhibitor (Sigma-Aldrich, P8340) and phosphatase inhibitor (PhosSTOP Tablets, Sigma-Aldrich). Plasma samples and lung homogenates were assayed for CXCL12 concentration using the mouse CXCL12/SDF1α Quantikine ELISA (catalog no. MCX120; R&D Systems, Minneapolis, MN, United States) according to the instruction of the manufacturer. The method was monitored using quality control samples provided in the assay kit. Mouse CXCL12 levels in BAL supernatant were determined using a commercially available electrochemiluminescence sandwich immunoassay (K152VBK-1; U-plex mouse CXCL12; Meso Scale Discovery). Recombinant human CXCL12 standard DuoSet kit (DY350; RnD Systems) was used for the standard curve. The assay was performed according to the instruction for use.

Mouse CXCL11 was quantified using an ultrasensitive immunoassay built on the Single Molecule Counting (SMC™) technology (Erenna® Immunoassay System, Merck Millipore, Billerica, MA, United States). Paramagnetic microparticles (beads) coated with anti-mouse CXCL11 monoclonal antibody (MAB572; R&D Systems) was used as the capturing antibody. Recombinant murine CXCL11 (250-29; Peprotech, Cranbury, NJ, United States) was used as a standard, and Fluor-labeled anti-mouse CXCL11 polyclonal antibody (AF572; R&D Systems) was used as the detection antibody. The number of fluorescently labeled detection antibodies counted with the Erenna® System is directly proportional to the amount of mouse CXCL11 present in the EDTA Plasma.

### CXCL9 and CXCL10 Measurement

Concentrations of the chemokines CXCL9 and CXCL10 in BAL supernatant were measured using a commercial mouse cytokine magnetic bead multiplex immunoassay kit (#MCYTOMAG-70K, Merck Millipore) according to the instruction of the manufacturer. Samples were acquired on a Luminex 200 instrument system (Thermo Fischer Scientific), and data were analyzed using SoftMaxPro software (Molecular Device, San Jose, CA, USA).

### Bronchoalveolar Lavage Supernatant Proteins

Protein concentrations in the BAL supernatant were determined using the BCA Protein assay kit (Pierce #23225, Thermo Fisher Scientific) according to the instruction of the manufacturer.

### Statistical Analysis

All data were expressed as mean ± standard error of the mean (SEM). Statistical analysis was performed using Prism version 8.1.1 (GraphPad software, San Diego, CA, United States) using the tests specified in the figure legends. Briefly, data were evaluated using two-tailed unpaired Student *t*-test, one-way or two-ways analysis of variance (ANOVA) with a *post-hoc* multiple comparison tests as appropriate. Differences were considered significant at *p* values <0.05.

## Results

### Lipopolysaccharide-Induced Acute Lung Injury/Acute Respiratory Distress Syndrome Model in DBA/1 Mice

Characterization of the consequences of LPS inhalation in DBA/1 mice was conducted to confirm in this experimental animal model (Matute-Bello et al., 2008) the presence of key features defining human ALI/ARDS, namely, breathing dysfunction, increased alveolar capillary barrier permeability, and immune cell infiltrates into the airspaces and the lung tissue.

**FIGURE 1:**
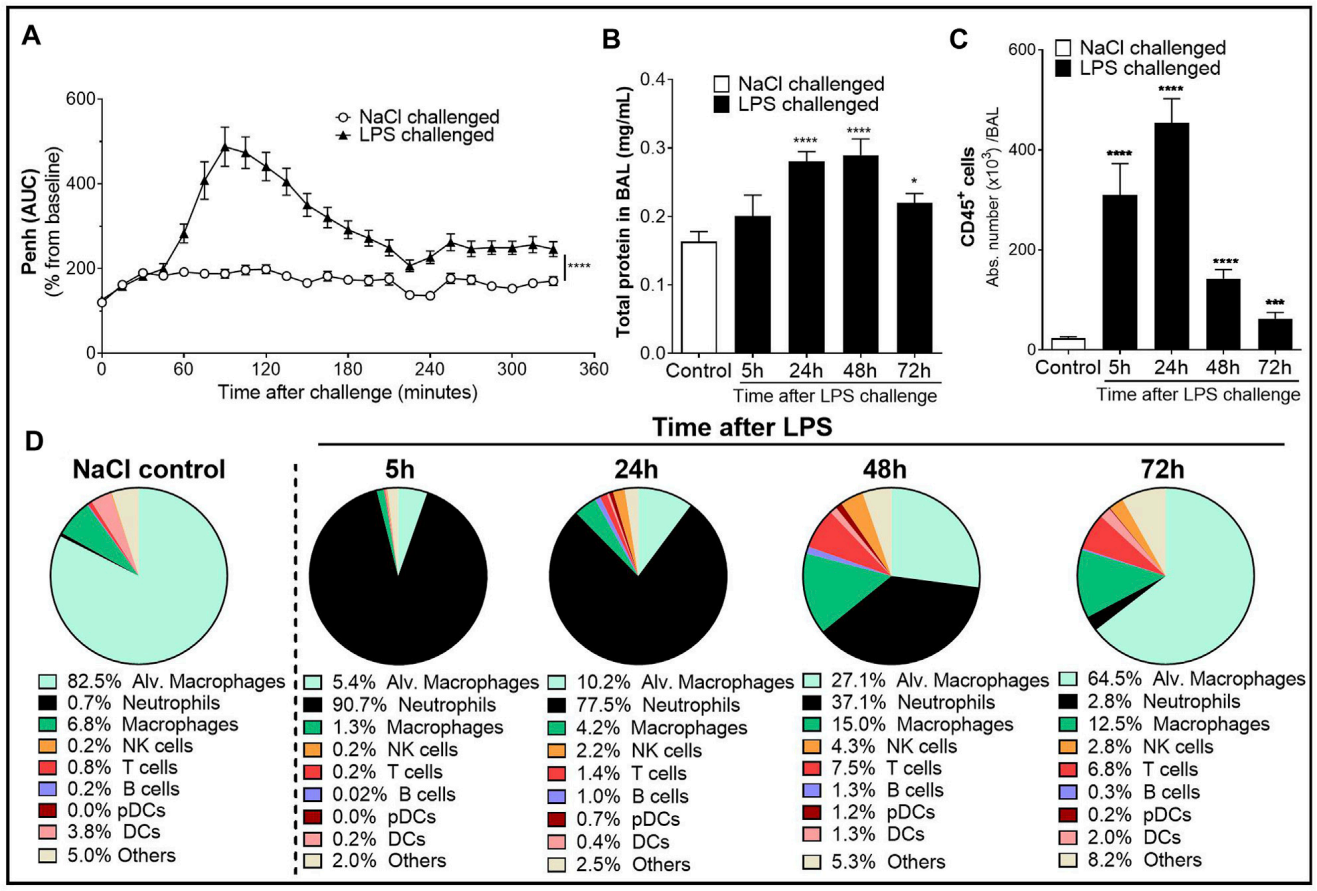
Characterization of the lipopolysaccharide (LPS)-induced acute lung injury (ALI)/acute respiratory distress syndrome (ARDS) model in male DBA/1 mice. LPS inhalation was associated with a significant breathing pattern alteration **(A)**, an increase in alveolar–capillary barrier permeability **(B)**, and increased immune cell infiltrates in the bronchoalveolar space **(C, D)** compared with NaCl-exposed mice (control mice). **(A)** Breathing pattern alteration was measured by the change in the calculated enhanced pause (Penh) using whole-body plethysmography in conscious unrestrained mice over a period of 6 h following LPS (black triangles) or NaCl inhalation (white circles). Results are expressed as the mean percentage Penh area under the curve (AUC) normalized to the baseline ± SEM (*n* = 16 mice per group). ****p < 0.0001 paired Student *t*-test. **(B)** Alveolar–capillary barrier permeability was assessed by measuring the total protein concentration in the bronchoalveolar lavage (BAL) supernatant at 5, 24, 48, and 72 h after LPS challenge (black bars, *n* = 7–16 mice per time point) or NaCl challenge (control mice, white bar, *n* = 20; all time points were pooled). Results are expressed as mean ± SEM. *p < 0.05, ****p < 0.0001 paired Student *t*-test. **(C)** Time course of CD45^+^ immune cells in the BAL measured by flow cytometry in samples collected at 5, 24, 48, and 72 h after LPS challenge (black bars, *n* = 7–16 mice per time point) or NaCl challenge (control mice, white bar, *n* = 20; all time points were pooled). Results are expressed as mean ± SEM. ***p < 0.001, ****p < 0.0001 paired Student *t*-test. **(D)** Frequencies of BAL immune cell populations over time. Results are expressed as proportions among total CD45^+^ cells for each time point.

Whole-body plethysmography on conscious, unrestrained mice was performed to monitor the physiological lung dysfunction caused by LPS. Inhalation of nebulized LPS caused a significant increase in enhanced pause (Penh), an index used as a marker of breathing pattern alteration (Hamelmann et al., 1997; Prada-Dacasa et al., 2020), compared with control mice receiving NaCl nebulization (Figure 1A). The increase in Penh peaked between 1.5 and 2 h after LPS challenge and remained elevated throughout the 6-h evaluation period.

To assess the effect of LPS on alveolar capillary barrier permeability, total protein concentration was measured in the BAL supernatant up to 72 h after LPS challenge. Total BAL protein concentration was significantly increased from 24 h up to 72 h after LPS challenge, compared with control mice (Figure 1B). To determine the effect of LPS on inflammatory cell recruitment to the alveolar space, BAL immune cell phenotyping was performed using flow cytometry. LPS inhalation led to a time-dependent infiltration of CD45^+^ immune cells into the BAL compared with control mice, peaking 24 h after LPS challenge (Figure 1C and Supplementary Figure S1). In control mice, the main BAL immune cell population was represented by tissue resident alveolar macrophages (Figure 1D and Supplementary Figure S1). In contrast, in LPS-exposed mice, 5 h after LPS challenge, neutrophils represented the majority of BAL immune infiltrates (Figure 1D). This early massive influx of neutrophils peaked 24 h after the LPS challenge before strongly decreasing 48 and 72 h post-LPS challenge (Table 1). Other BAL immune cells such as NK cells, B cells, pDCs, and classical CD11b^−^ DCs also peaked 24 h after LPS challenge (Table 1) but altogether represented less than 9% of the overall BAL population at any given time point (Figure 1D). In contrast, infiltration of inflammatory macrophages and T cells peaked later, at 48 h after LPS challenge (Table 1), and together represented more than 20% of the BAL immune cells (Figure 1D). At 72 h post-LPS challenge, BAL immune cell numbers were still significantly increased compared with control mice (Figure 1C and Table 1). Infiltration of neutrophils was also confirmed in the lung tissue by histology, 24 h after LPS challenge (Supplementary Figure S2).

**TABLE 1:** Time course of immune cells present in the bronchoalveolar lavage (BAL) following LPS or NaCl challenge in DBA/1 mice.

Challenge:		NaCl (control)	Lipopolysaccharide (LPS)			
Time points post challenge		Pool (*n* = 20)	5 h (*n* = 8)	24 h (*n* = 16)	48 h (*n* = 16)	72 h (*n* = 7–8)
			Abs number (×10^3^ cells/BAL)			
CD11b^low^ FSC^high^	Alveolar macrophages	19.4 ± 2.9	16.6 ± 2.7	46.3 ± 13.1*	38.7 ± 10.2	40.5 ± 9.6*
CD11b^+^ cells (Myeloid cells)	Neutrophils	0.2 ± 0.0	281.0 ± 61.4****	352.3 ± 29.2****	53.0 ± 8.3****	1.7 ± 0.3****
	Inflammatory macrophages	1.6 ± 0.3	4.1 ± 0.5***	19.1 ± 3.6****	21.4 ± 3.1****	7.8 ± 1.4****
CD11b^−^ cells (Lymphoid cells)	NK cells	0.1 ± 0.0	0.6 ± 0.1****	9.9 ± 1.6****	6.1 ± 1.1****	1.7 ± 0.4****
	T cells	0.2 ± 0.0	0.6 ± 0.1***	6.2 ± 0.8****	10.7 ± 2.1****	4.3 ± 0.9****
	B cells	0.0 ± 0.0	0.1 ± 0.0	4.6 ± 1.6**	1.8 ± 0.4****	0.2 ± 0.0*
	pDCs	0.0 ± 0.0	0.0 ± 0.0	3.4 ± 0.9***	1.7 ± 0.6**	0.1 ± 0.0****
	DCs	0.9 ± 0.2	0.7 ± 0.0	1.8 ± 0.4*	1.8 ± 0.2**	1.3 ± 0.2

Note. Acute lung injury (ALI) was induced by LPS nebulization in male DBA/1 mice, and flow cytometry analysis of BAL immune infiltrates was performed 5, 24, 48, and 72 h after LPS challenge (*n* = 7–16 mice per time point). Control mice received a nebulization of NaCl 0.9% (*n* = 20 mice; all time points were pooled). The gating strategy for all immune populations is illustrated in Supplementary Figure S1A. Results are expressed as mean ± SEM.

*p < 0.05, **p < 0.01, ***p < 0.001, ****p < 0.0001 versus control mice using Student *t*-test.

Taken together, these results demonstrate that inhalation of nebulized LPS in DBA/1 mice induced an acute and time-dependent increase in breathing dysfunction, vascular permeability, and infiltration of immune cells into the alveolar space and the lung tissue, and confirm this model as suitable to evaluate these pathogenic features of human ALI/ARDS in DBA/1 mice.

### The Increase of CXCR3 and CXCR4 Ligands in the Bronchoalveolar Lavage is Associated With an Increase of CXCR3^+^ and CXCR4^+^ Bronchoalveolar Lavage Immune Cell Infiltrates

To confirm that in this strain of mice both the CXCR3/CXCL9/10/11 and CXCR4/CXCL12 axes played a role in the recruitment of immune infiltrates into the BAL, the expression of CXCR3 and CXCR4 on infiltrating immune cells and the release of their ligands were characterized in the alveolar space following LPS challenge.

**FIGURE 2:**
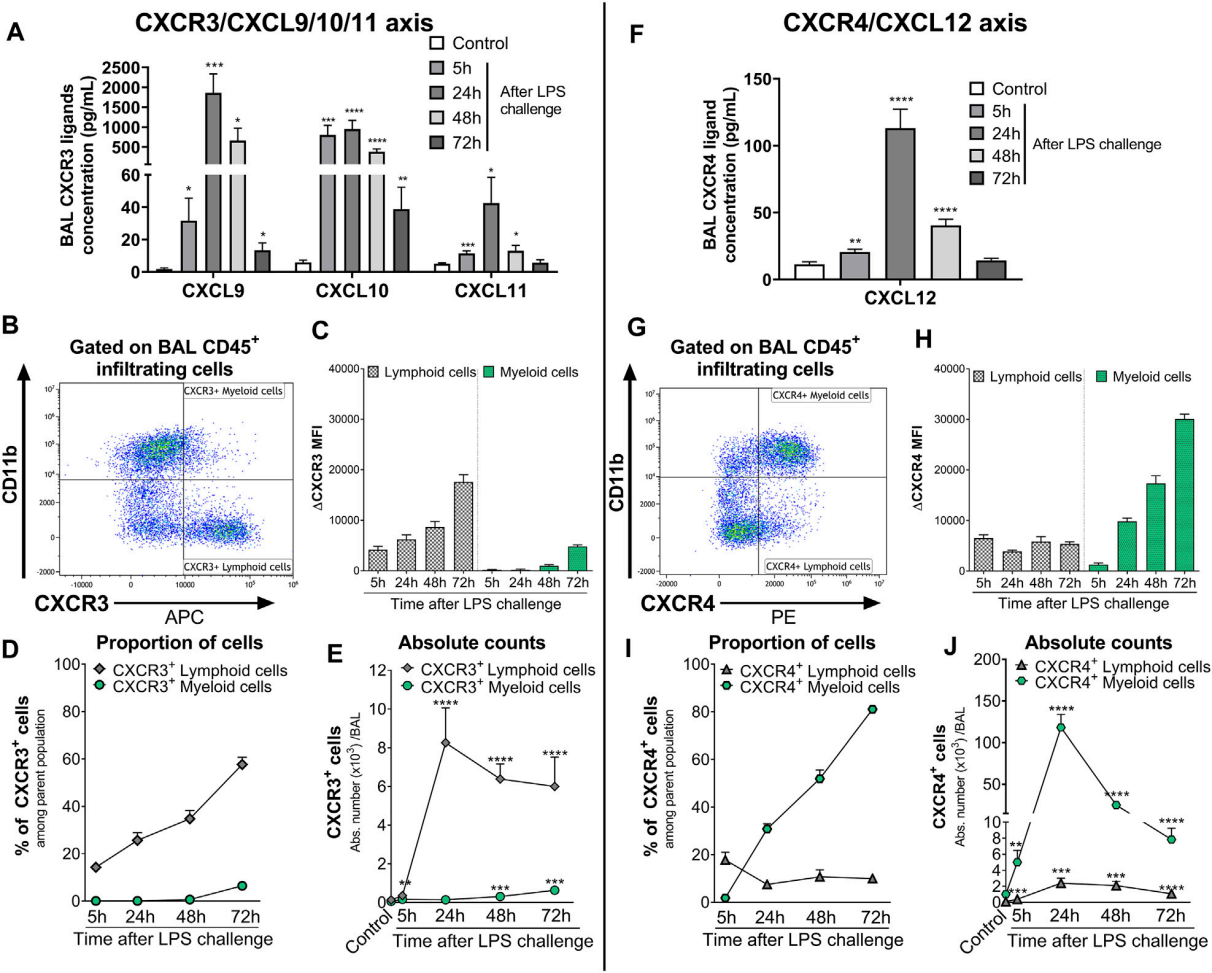
Kinetics of the expression of CXCR3/CXCR4 and its ligands in the BAL of LPS-challenged DBA/1 mice. ALI was induced by nebulized LPS inhalation in male DBA/1 mice. Control mice inhaled NaCl 0.9% (*n* = 12 mice; all time points were pooled). **(A)** LPS inhalation increased protein concentrations of the CXCR3 ligands CXCL9, CXCL10, and CXCL11, measured in the BAL 5, 24, 48, and 72 h following LPS challenge, compared with control mice. Results are expressed as mean ± SEM (*n* = 8 mice per time point). *p < 0.05, **p < 0.01, ***p < 0.001, ****p < 0.0001 versus control mice using Student *t*-test. **(B)** Representative gating strategy for CXCR3^+^ myeloid and lymphoid cells. **(C)** CXCR3 expression on BAL lymphoid and myeloid-infiltrating cells. Results are expressed as mean ± SEM of the mean fluorescence intensity (MFI) of CXCR3 obtained for each LPS-challenged mouse (*n* = 7–8 mice per time point) corrected by the MFI obtained in the fluorescence minus one (FMO) controls for CXCR3. Negative MFI values were set to 0. The complete gating strategy for lymphoid and myeloid cells is illustrated in Supplementary Figure S3. **(D)** Proportion of CXCR3^+^ lymphoid and myeloid cells expressed as percentages (mean ± SEM) of the CD45^+^CD11b^−^ and CD45^+^CD11b^+^ parent population, respectively, in the BAL (see gating strategy in Supplementary Figure S3) (*n* = 7–8 mice per time point). **(E)** Time course of BAL CXCR3^+^ lymphoid and myeloid cell infiltrates. Results are expressed as absolute counts in the BAL (mean ± SEM). **p < 0.01, ***p < 0.001, ****p < 0.0001 using Student *t*-test versus control mice. **(F)** LPS inhalation increased protein concentrations of the CXCR4 ligand CXCL12, measured in the BAL 5, 24, 48, and 72 h following LPS nebulization compared with control mice. Results are expressed as mean ± SEM (*n* = 7–8 mice per time point). **p < 0.01, ****p < 0.0001 versus control mice using Student *t*-test. **(G)** Representative gating strategy for CXCR4^+^ myeloid and lymphoid cells. **(H)** CXCR4 expression on BAL lymphoid and myeloid-infiltrating cells. Results are expressed as mean ± SEM of the MFI of CXCR4 obtained for each LPS-challenged mouse (*n* = 7–8 mice per time point) corrected by the MFI obtained in the FMO controls for CXCR4. Negative MFI values were set to 0. The gating strategy for lymphoid and myeloid cells is illustrated in Supplementary Figure S3. **(I)** Proportion of CXCR4^+^ lymphoid and myeloid cells expressed as percentages (mean ± SEM) of the CD45^+^CD11b^−^ and CD45^+^CD11b^+^ parent population, respectively, in the BAL (see gating strategy in Supplementary Figure S3) (*n* = 7–8 mice per time point). **(H)** Time course of BAL CXCR4^+^ lymphoid and myeloid cell infiltrates. Results are expressed as absolute counts in the BAL (mean ± SEM) (*n* = 7–8 mice per time point). **p < 0.01, ***p < 0.001, ****p < 0.0001 using Student *t*-test versus control mice.

In the BAL supernatant, LPS challenge led to a significant increase in the three CXCR3 ligands CXCL9, CXCL10, and CXCL11, compared with control mice (Figure 2A), reaching the highest increase 24 h post-LPS challenge. Seventy-two hours after LPS challenge, CXCL11 returned to control levels, while CXCL9 and CXCL10 were still significantly elevated compared with control mice (Figure 2A). CXCR3 expression was mainly detected on CD11b^−^ BAL lymphoid cells compared with CD11b^+^ BAL myeloid cells; in the latter population, CXCR3 was only detected at low levels 48 and 72 h post LPS challenge (Figures 2B, C and Supplementary Figure S3). Interestingly, the expression of CXCR3 (Figure 2C) and proportion of CXCR3^+^ BAL lymphoid and myeloid cells (Figure 2D) increased over time following LPS nebulization. At 72 h post LPS challenge, almost 60% of BAL lymphoid cells expressed CXCR3 (Figure 2D); in absolute counts, CXCR3^+^ BAL lymphoid cells were only slightly reduced compared with earlier time points but were still significantly increased compared with control mice (Figure 2E).

LPS inhalation also led to a significant increase in BAL CXCL12 levels compared with control mice, which peaked at 24 h and returned to control levels 72 h post-LPS challenge (Figure 2F). In the BAL from LPS-challenged mice, CXCR4 expression was detected on the cell surface of most infiltrating leukocytes, with the highest surface expression detected on myeloid cells (Figure 2G and Supplementary Figure S3). While the expression of CXCR4 on lymphoid cells and the proportion of CXCR4^+^ BAL lymphoid infiltrates remained stable following LPS nebulization, the expression of CXCR4 on myeloid cells and the proportion of CXCR4^+^ BAL myeloid infiltrates increased over time, reaching over 80% at the last time point investigated (Figures 2H, I). In absolute counts, the highest number of CXCR4^+^ BAL myeloid cells was reached 24 h post-LPS challenge but still remained significantly elevated 72 h post-LPS challenge, compared with control mice (Figure 2J).

In conclusion, LPS challenge of DBA/1 mice resulted in a significant increase in CXCR3/CXCR4 ligand levels, including the CXCR7 ligands CXCL11 and CXCL12, in the BAL supernatant. This increase was associated with a significant increase in predominantly CXCR3^+^ lymphoid and CXCR4^+^ myeloid cells in the BAL.

### CXCR7 Antagonism Increases Plasma CXCL11 and CXCL12 Levels and is Associated With a Reduction in CXCR3^+^ and CXCR4^+^ Bronchoalveolar Lavage Immune Cell Infiltrates After Lipopolysaccharide Challenge

The scavenging activity of CXCR7 expressed on endothelial cells has been proposed to generate CXCL11 and CXCL12 chemokine gradients, thus, enabling a directional migration of CXCR3^+^ and CXCR4^+^ cells, respectively, from the circulation toward sites of inflammation (Berahovich et al., 2014; Tobia et al., 2019; Pouzol et al., 2021). To evaluate this hypothesis, the impact of CXCR7 antagonism on plasma and lung tissue CXCL11 and CXCL12 levels and BAL immune cell infiltrates was investigated in the ALI/ARDS DBA/1 mouse model following treatment with ACT-1004-1239.

**FIGURE 3:**
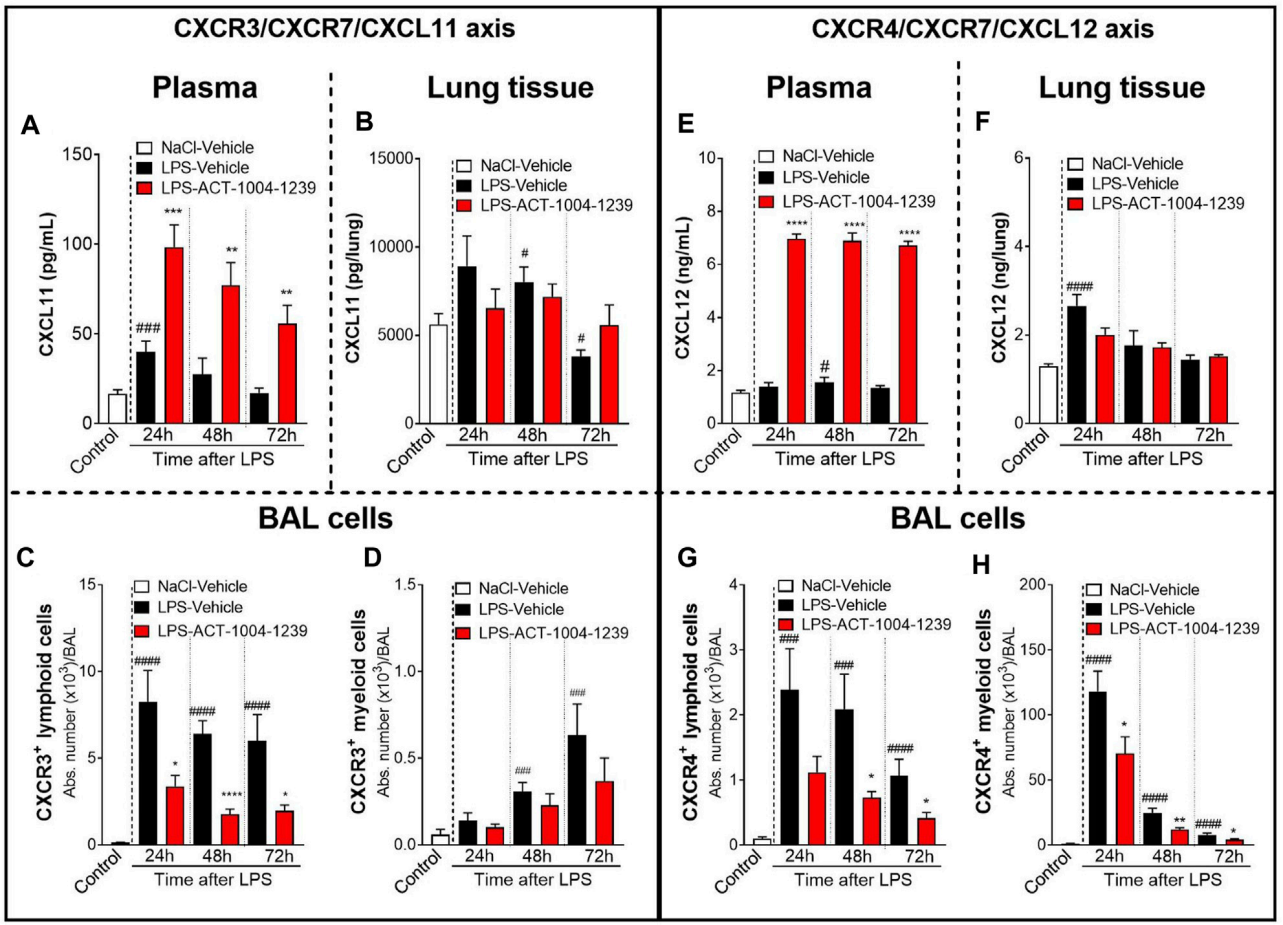
Antagonism of CXCR7 increases plasma CXCL11 and CXCL12 levels and decreases CXCR3^+^ and CXCR4^+^ BAL infiltrates post LPS challenge. ALI was induced by nebulized LPS inhalation, and DBA/1 mice were treated with vehicle (LPS-vehicle, black bars) or ACT-1004-1239 (LPS-ACT-1004-1239, 100 mg/kg, red bars) orally, twice daily, 1 h prior to LPS challenge. Control mice received vehicle 1 h prior to NaCl 0.9% inhalation (NaCl-vehicle, white bars; *n* = 12 mice, pool of all time points). Protein concentrations of CXCL11 and CXCL12 in the plasma and lung tissue and BAL flow cytometry of immune infiltrates were performed 24, 48, and 72 h after challenge (*n* = 6–8 mice per time point). Time course of CXCL11 protein concentration in the plasma **(A)** and lung tissue **(B)**. Chemokine concentrations are expressed in pg/ml (plasma) or pg/lung homogenate (mean ± SEM). **p < 0.01, ***p < 0.001 versus LPS-vehicle-treated animals or ^#^ *p* < 0.05, ^###^ *p* < 0.001 versus NaCl-vehicle-treated control mice using Student *t*-tests. Time course of BAL CXCR3^+^ lymphoid **(C)** and myeloid **(D)** infiltrates. Results are expressed as absolute cell counts in the BAL (mean ± SEM). *p < 0.05, ****p < 0.0001 versus LPS-vehicle-treated animals or ^###^ *p* < 0.001, ^####^ *p* < 0.0001 versus NaCl-vehicle-treated control mice using Student t-tests. Time course of CXCL12 concentration in the plasma **(E)** and lung tissue **(F)**. Chemokine concentrations are expressed in ng/ml (plasma) or ng/lung homogenate (mean ± SEM). ****p < 0.0001 versus LPS-vehicle-treated animals or ^#^ *p* < 0.05, ^###^ *p* < 0.001 versus NaCl-vehicle-treated control mice using Student t-tests. Time course of BAL CXCR4^+^ lymphoid **(G)** and BAL CXCR4^+^ myeloid **(H)** infiltrates. Results are expressed as absolute counts in the BAL (mean ± SEM). *p < 0.05, **p < 0.01, using Student t-test versus LPS-vehicle-treated animals or ^###^ *p* < 0.001, ^####^ *p* < 0.0001 versus NaCl-vehicle-treated control mice using Student t-tests.

CXCR7 antagonism further increased the LPS-induced elevation of CXCL11 plasma levels at all time points investigated compared with vehicle-treated, LPS-challenged mice (Figure 3A), confirming the proposed scavenging activity of CXCR7. In contrast, in the lung tissue, treatment with ACT-1004-1239 did not further increase CXCL11 concentrations but rather tended to normalize CXCL11 levels from LPS-challenged mice throughout the study period (Figure 3B). In the BAL from LPS-challenged mice, treatment with ACT-1004-1239 significantly reduced LPS-induced CXCR3^+^ lymphoid cell recruitment (Figure 3C) and showed a trend to decrease the late recruitment of the few CXCR3^+^ myeloid cells induced by LPS, although this did not reach statistical significance (Figure 3D).

Treatment with ACT-1004-1239 led to a robust and significant increase in plasma CXCL12 concentrations at all time points tested compared with vehicle-treated, LPS-challenged mice (Figure 3E). In contrast to the effect seen in plasma, in the lung tissue, CXCR7 antagonism did not further increase the LPS-induced elevation in CXCL12 levels and even resulted in a slight reduction in CXCL12 levels at 24 h post LPS challenge compared with vehicle-treated LPS-challenged mice (Figure 3F). In the BAL from LPS-challenged mice, at all time points investigated, treatment with ACT-1004-1239 significantly reduced BAL CXCR4^+^ lymphoid (Figure 3G) and CXCR4^+^ myeloid cell infiltrates (Figure 3H) compared with vehicle-treated LPS-challenged mice.

**FIGURE 4:**
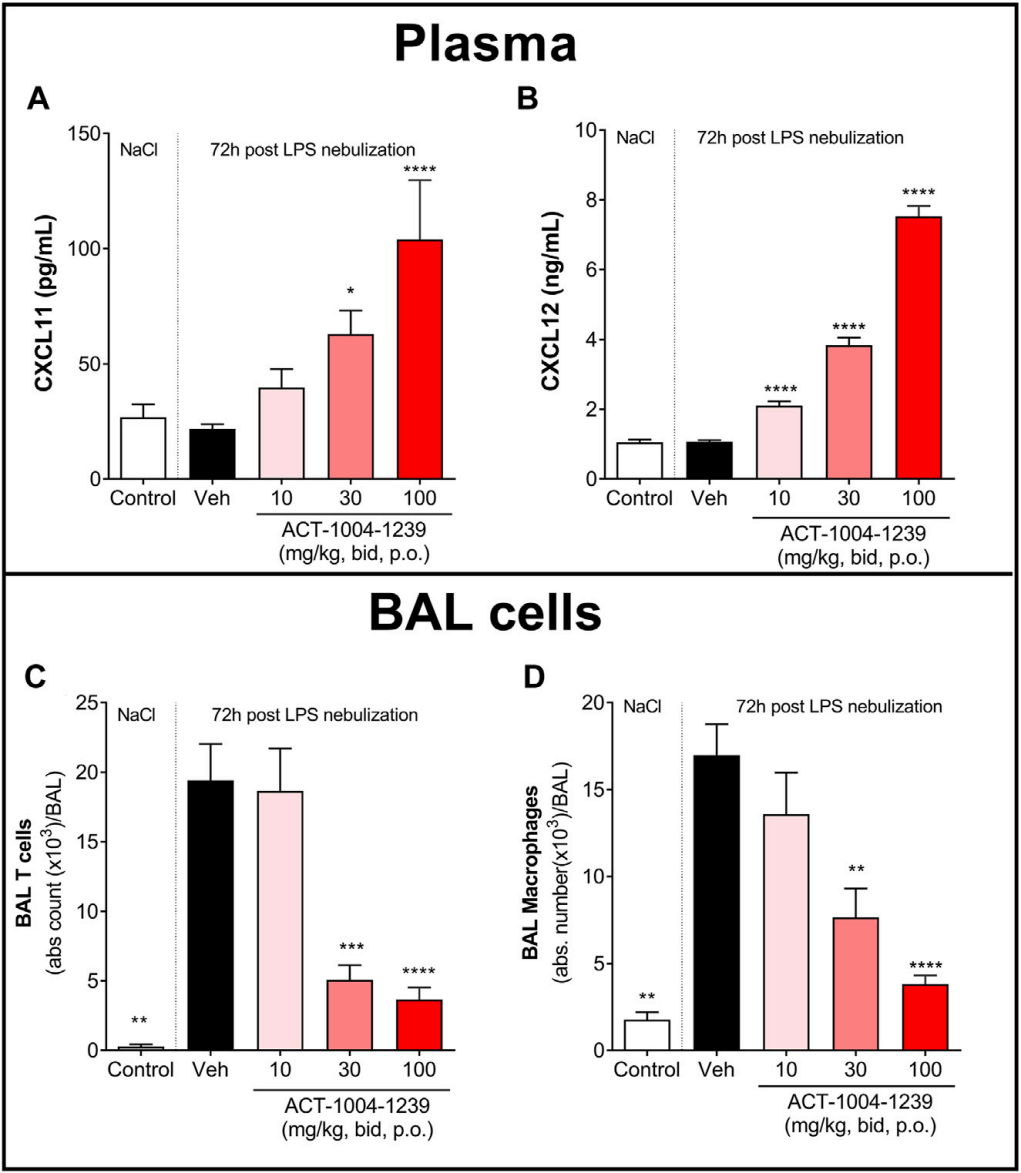
Treatment with ACT-1004-1239 dose-dependently increases plasma CXCL11 and CXCL12 levels and reduces BAL T cell and inflammatory macrophage infiltrates in the LPS-induced ALI/ARDS model. Vehicle (Veh; black bars) or ACT-1004-1239 (10, 30, or 100 mg/kg; bars with different shades of red) was given orally, twice daily, starting 1 h prior to LPS nebulization, for a total of six administrations. Control mice (white bars) were challenged by NaCl nebulization and received vehicle administrations. Plasma CXCL11 **(A)** and plasma CXCL12 levels **(B)** 72 h after LPS or NaCl challenge. Results are expressed as mean + SEM (*n* = 10–25 mice per treatment-LPS groups and *n* = 4–5 mice for control group). *p < 0.05, ****p < 0.0001 versus vehicle-treated LPS-challenged mice, using one-way ANOVA test followed by Dunnett’s multiple comparisons test. Total BAL T cell **(C)** and BAL inflammatory macrophage counts **(D)** 72 h after LPS challenge. Results are expressed as mean ± SEM with *n* = 11–23 mice per treatment-LPS groups and *n* = 3 for controls. **p < 0.01, ***p < 0.001, ****p < 0.0001 versus vehicle-treated LPS-challenged mice, using one-way ANOVA test followed by Dunnett’s multiple comparisons test.

To investigate the dose-dependent effect of CXCR7 antagonism on plasma CXCL11 and CXCL12 levels and on immune cell recruitment to the alveolar space, mice were treated with three different doses of ACT-1004-1239 (10, 30, and 100 mg/kg, p.o., twice daily) or vehicle for 3 days, starting 1 h prior to LPS challenge. Treatment with ACT-1004-1239 dose-dependently increased plasma CXCL11 and CXCL12 concentrations (Figures 4A, B); this was accompanied by a dose-dependent decrease in the major immune cell infiltrates present in the BAL after 72 h (Figure 4D), namely, T cells (Figure 4C) and macrophages (Figure 4D).

Taken together, these data demonstrate that CXCR7 antagonism with ACT-1004-1239 significantly increased plasma CXCL11 and CXCL12 levels in a dose-dependent manner and was associated with a reduction in CXCR3^+^ and CXCR4^+^ BAL immune infiltrates.

### CXCR7 Antagonism Reduces Lipopolysaccharide -Induced Acute Lung Injury/Acute Respiratory Distress Syndrome

**FIGURE 5:**
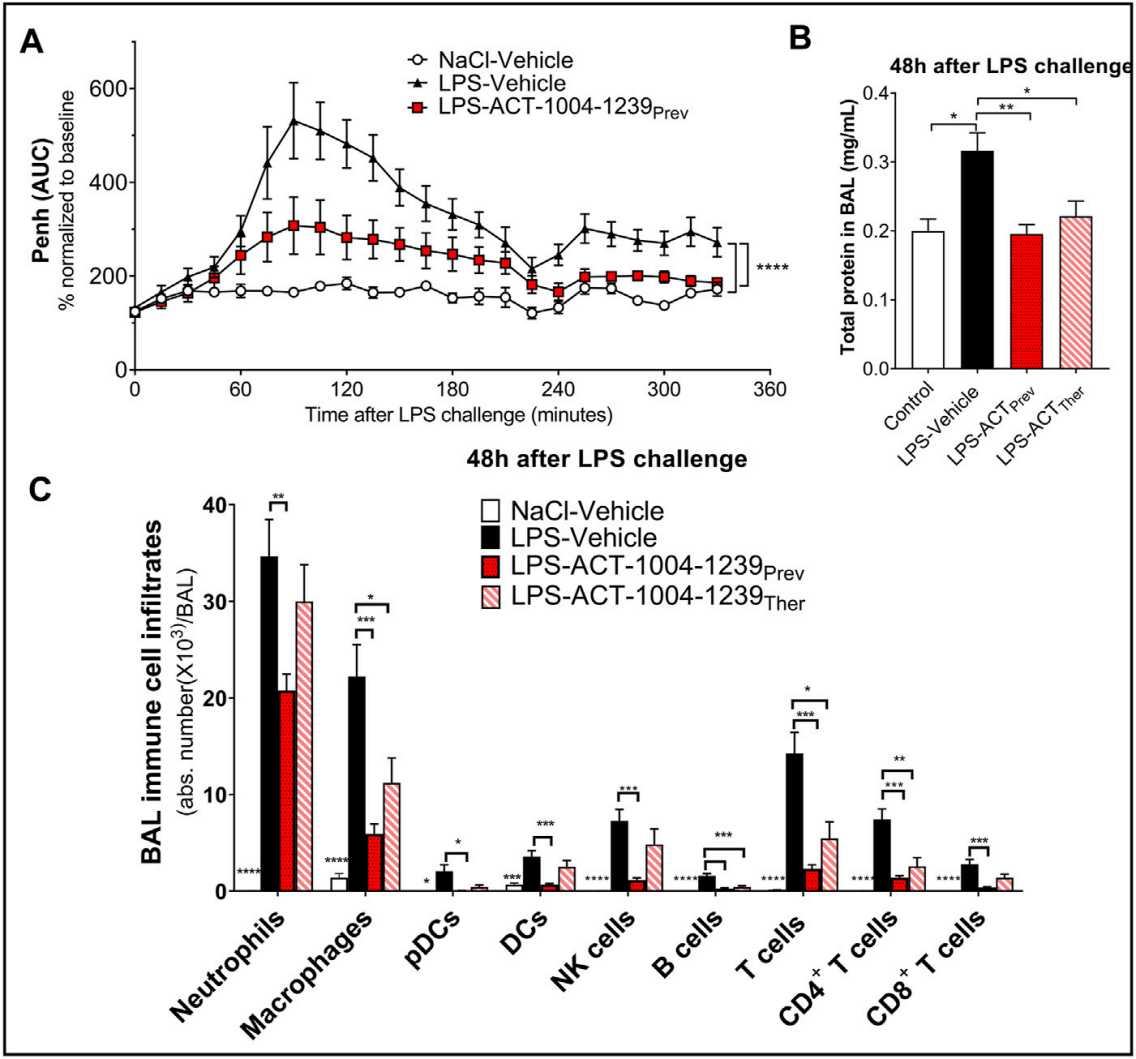
Antagonism of CXCR7 reduces LPS-induced ALI/ARDS. Treatment with the CXCR7 antagonist ACT-1004-1239 significantly reduced LPS-induced breathing pattern alteration **(A)**, alveolar–capillary barrier permeability **(B)**, and immune cell infiltrates in the bronchoalveolar space **(C)** compared with vehicle-treated-LPS exposed mice. Mice were treated with vehicle (LPS-vehicle; black) or ACT-1004-1239 (100 mg/kg, LPS-ACT-1004-1239) orally, twice daily, starting 1 h prior to LPS challenge (preventive setting; LPS-ACT_Prev_; red) or 3 h after LPS challenge (therapeutic setting; LPS-ACT_Ther_; hatched bar). Control mice were treated with vehicle and inhaled NaCl 0.9% (NaCl-vehicle; white). **(A)** Preventive treatment with ACT-1004-1239 reduced the LPS-induced breathing pattern alteration, measured by the calculated enhanced pause (Penh) using whole-body plethysmography in conscious unrestrained mice over a period of 6 h following LPS inhalation. Results are expressed as the mean percentage Penh area under the curve (AUC) normalized to the baseline ± SEM (*n* = 8 mice per group). ****p < 0.0001 using two-way ANOVA, followed by Dunnett’s multiple comparisons test versus LPS-vehicle-treated mice. **(B)** Preventive and therapeutic treatment with ACT-1004-1239 reduced alveolar–capillary barrier permeability, measured by a reduction in total protein concentration in BAL supernatant 48 h after LPS challenge (*n* = 7–16 mice per LPS-challenged groups; *n* = 6 NaCl-vehicle control mice). Results are expressed as mean + SEM. *p < 0.05, **p < 0.01 versus LPS-vehicle-treated mice using one-way ANOVA followed by Dunnett’s multiple comparisons test. **(C)** Preventive and therapeutic treatment with ACT-1004-1239 reduced immune cell infiltrates in the BAL, measured by flow cytometry 48 h after LPS challenge (*n* = 7–16 mice per LPS-challenged groups; *n* = 6 NaCl-vehicle control mice). Results are expressed as mean ± SEM. *p < 0.05, **p < 0.01, ***p < 0.001, ****p < 0.0001 versus LPS-vehicle-treated mice using two-way ANOVA followed by Dunnett’s multiple comparisons test. The gating strategy for all immune populations is illustrated in Supplementary Figures S1A,B.

To monitor whether the CXCR7 antagonist ACT-1004-1239 would affect LPS-induced breathing dysfunction, whole-body plethysmography on conscious, unrestrained DBA/1 mice was performed. As previously reported (Lefort et al., 2001), LPS nebulization caused a significant increase in enhanced pause (Penh) AUC versus vehicle-treated mice challenged with NaCl (Figure 5A). A single oral administration of ACT-1004-1239 (100 mg/kg, p.o.), given 1-h prior to LPS challenge, significantly reduced the LPS-induced elevated Penh AUC, reaching values from control mice at the end of the evaluation period (Figure 5A), indicating a normalization of the breathing pattern. In addition, the effect of CXCR7 antagonism on LPS-induced alveolar capillary barrier permeability increase was assessed 48 h post-LPS challenge, at the peak of the LPS effect (Figure 1B). Treatment with ACT-1004-1239 (100 mg/kg, p.o., twice daily) was initiated either 1-h prior to LPS challenge (preventive setting) or 3 h after LPS challenge (therapeutic setting), when neutrophil infiltration was already apparent (data not shown). In both settings, treatment with the CXCR7 antagonist significantly reduced the overall protein content in the BAL, compared with vehicle-treated, LPS-challenged mice (Figure 5B). Moreover, treatment with ACT-1004-1239 reduced LPS-induced leukocyte recruitment to the BAL 48 h post-LPS challenge. Preventive ACT-1004-1239 treatment significantly reduced all evaluated immune cell subsets present in the BAL (Figure 5C), whereas therapeutic administration of ACT-1004-1239 significantly reduced macrophages and lymphocytes, and showed a trend to reduce all other evaluated cell subtypes without reaching statistical significance (Figure 5C).

In summary, blockade of the CXCR7 axis improved clinical signs of nebulized LPS inhalation as shown by an improvement in the breathing pattern, a reduction in the vascular barrier dysfunction, and a reduction in immune cell infiltrates into the BAL, thus, confirming the importance of the CXCR3/CXCR4/CXCR7 axis in ALI/ARDS.

## Discussion

Acute lung injury and its more severe form, ARDS, represent lung disease conditions of multifactorial etiology, associated with diffuse alveolar damage and hypoxemia. Despite better knowledge regarding the pathogenesis of ALI/ARDS, mortality remains high (40%), and current treatment is restricted to supportive care with mechanical ventilation, emphasizing the need to develop and test new therapies for this life-threatening condition (Dushianthan et al., 2011; Bellani et al., 2016). The lack of effective therapy has been recently underlined by the coronavirus disease 19 (COVID-19) pandemic, which causes ARDS in 3%–5% of the patients infected with SARS-CoV-2 (Horie et al., 2020).

The preclinical ALI/ARDS model, induced by LPS inhalation in rodents, is a commonly used model, which manifests key features of human ALI/ARDS (Menezes et al., 2005; Matute-Bello et al., 2008). In the current study, the consequences of LPS inhalation were characterized over time in DBA/1 mice, a strain of mice expressing both CXCR7 ligands (CXCL11 and CXCL12). In line with previous reports in similar models but not expressing CXCL11 (Lefort et al., 2001; Lax et al., 2014), inhalation of nebulized LPS in DBA/1 mice induced key parameters recommended by the American Thoracic Society to detect the presence of ARDS in laboratory animals: LPS inhalation caused a rapid and significant recruitment of inflammatory cells to the alveolar space, especially neutrophils at early time points after LPS challenge followed by macrophages and T cells at later time points. Furthermore, LPS inhalation increased alveolar capillary barrier permeability. The LPS-induced breathing pattern alteration was also observed, which has been shown to be associated with altered lung function (Håkansson et al., 2012).

The duration of the pulmonary inflammatory contributes to the pathogenesis of ALI/ARDS and may determine the severity and subsequent mortality in patients (Meduri et al., 1995; Yang et al., 2003); however, the mechanisms leading to a persistent inflammation remain unclear. Previous reports have suggested that both the CXCR3 and CXCR4 axis play a pivotal role in the prolonged recruitment and/or retention of immune cells in ALI/ARDS, exerting a damaging effect in the lung (Petty et al., 2007; Nie et al., 2008; Kelsen et al., 2009).

Consistent with findings from human patients and mouse models of ALI (Saetta et al., 2002; Petty et al., 2007; Hartl et al., 2008; Costello et al., 2012; Ichikawa et al., 2013; Ngamsri et al., 2017; Liao et al., 2020), in this study using LPS-challenged DBA/1 mice, CXCR3 and CXCR4 ligands were elevated in the BAL, which was associated with increased BAL CXCR3^+^ and CXCR4^+^ cell infiltrates. In line with previously reported data (Petty et al., 2007), the expression of CXCR4 on BAL myeloid cells steadily increased over time following LPS-induced lung injury. Importantly, the same observation was made regarding the expression of CXCR3, especially on lymphoid cells, suggesting a distinct role for CXCR3 and CXCR4 in the recruitment and persistence/retention of BAL infiltrates during ALI/ARDS. Interestingly, a similar observation was made in the BAL from patients with COVID-19, where most BAL infiltrates expressed CXCR3 and/or CXCR4 (Liao et al., 2020), and the increased presence of activated lung-homing CXCR4^+^ T cells was associated with fatal COVID-19 (Neidleman et al., 2021).

CXCR7 has been reported to scavenge both CXCL11 and CXCL12. Even though CXCL11 was found at lower levels than CXCL9 and CXCL10 in the BAL from LPS challenged mice, this chemokine represents the most potent CXCR3 ligand (Sauty et al., 2001). To date, the role of the CXCR3/CXCR7/CXCL11 axis in preclinical ALI models has not been investigated as previous studies were conducted in C57BL/6 mice, a mouse strain lacking CXCL11 (Sierro et al., 2007). We show here that antagonizing CXCR7 with ACT-1004-1239 not only led to the elevation of CXCL12 in the plasma, confirming previous data (Richard-Bildstein et al., 2020; Pouzol et al., 2021) but also of the inducible chemokine CXCL11, in a dose-dependent manner, confirming the inhibition of the scavenging activity of CXCR7 *in vivo*. In contrast, CXCR7 antagonism did not further increase the LPS-induced elevation of CXCL11 and CXCL12 concentrations in the inflamed lung tissue, but rather tended to normalize them, likely leading to a disruption in the chemokine concentration gradient from the blood to the injured lung tissue. Consequently, a significant reduction in CXCR3^+^ lymphoid and CXCR4^+^ myeloid and lymphoid cells in the BAL was observed upon treatment with the CXCR7 antagonist, suggesting an inhibition of directional migration of these cells from the blood to the inflamed tissue and/or a reduction in their retention in the tissue. The immunomodulatory effect of CXCR7 antagonism was consistently shown both in a preventive and therapeutic setting, highlighting its potential clinical impact on acute pulmonary inflammation.

Besides the pivotal role of CXCR7 on immune cell infiltration to the BAL, expression of CXCR7 in the vasculature has been associated with a disruption of the endothelial barrier function (Totonchy et al., 2014). CXCR7 antagonism with ACT-1004-1239, both in a preventive and therapeutic setting, successfully reduced alterations in the alveolar capillary barrier function as shown by a reduction in the overall protein content in the BAL following LPS challenge. Furthermore, CXCL12 has been shown to promote endothelial barrier integrity *in vivo* via CXCR4 (Kobayashi et al., 2014) and to enhance barrier function in human pulmonary artery endothelial cells following thrombin activation *in vitro* (Cheng et al., 2017). These data suggest that the reduced vascular permeability seen in the ALI/ARDS model following CXCR7 antagonism could be a result of a direct effect on endothelial cells and/or indirect effect via increased CXCL12 concentration.

An additional interesting finding of this study was the effect of ACT-1004-1239 on breathing pattern alteration following LPS challenge. Endotoxins have been related to pulmonary functional disturbances both in humans (Leaker et al., 2013) and mice (Lefort et al., 2001) and have been shown to exacerbate established emphysema in several experimental models (Kobayashi et al., 2013; de Oliveira et al., 2019). Therefore, this aspect may be relevant to the pathogenesis of ARDS, especially at the late stages where emphysema-like lesions are present (Terzi et al., 2014). In the present study, treatment with ACT-1004-1239 reduced the Penh increase observed within 2 h following LPS inhalation. Published data in similar models reported that LPS-induced Penh increase was dependent on TNFα release (Schnyder-Candrian et al., 2005). However, the effect observed with ACT-1004-1239 on this early breathing pattern change could not be explained by a reduction in LPS-induced BAL TNFα elevation (data not shown). More studies are needed to unravel the mechanisms that account for this effect.

So far, only CCX771, which recruits β-arrestin upon binding to the CXCR7 receptor, has been evaluated in the context of ALI (Zabel et al., 2009; Ngamsri et al., 2017). Our study using ACT-1004-1239, a CXCR7 antagonist of the β-arrestin pathway, provides evidence that the observed efficacy obtained with CCX771 in ALI was likely due to its functional antagonistic effect and not to its agonistic effect.

**FIGURE 6:**
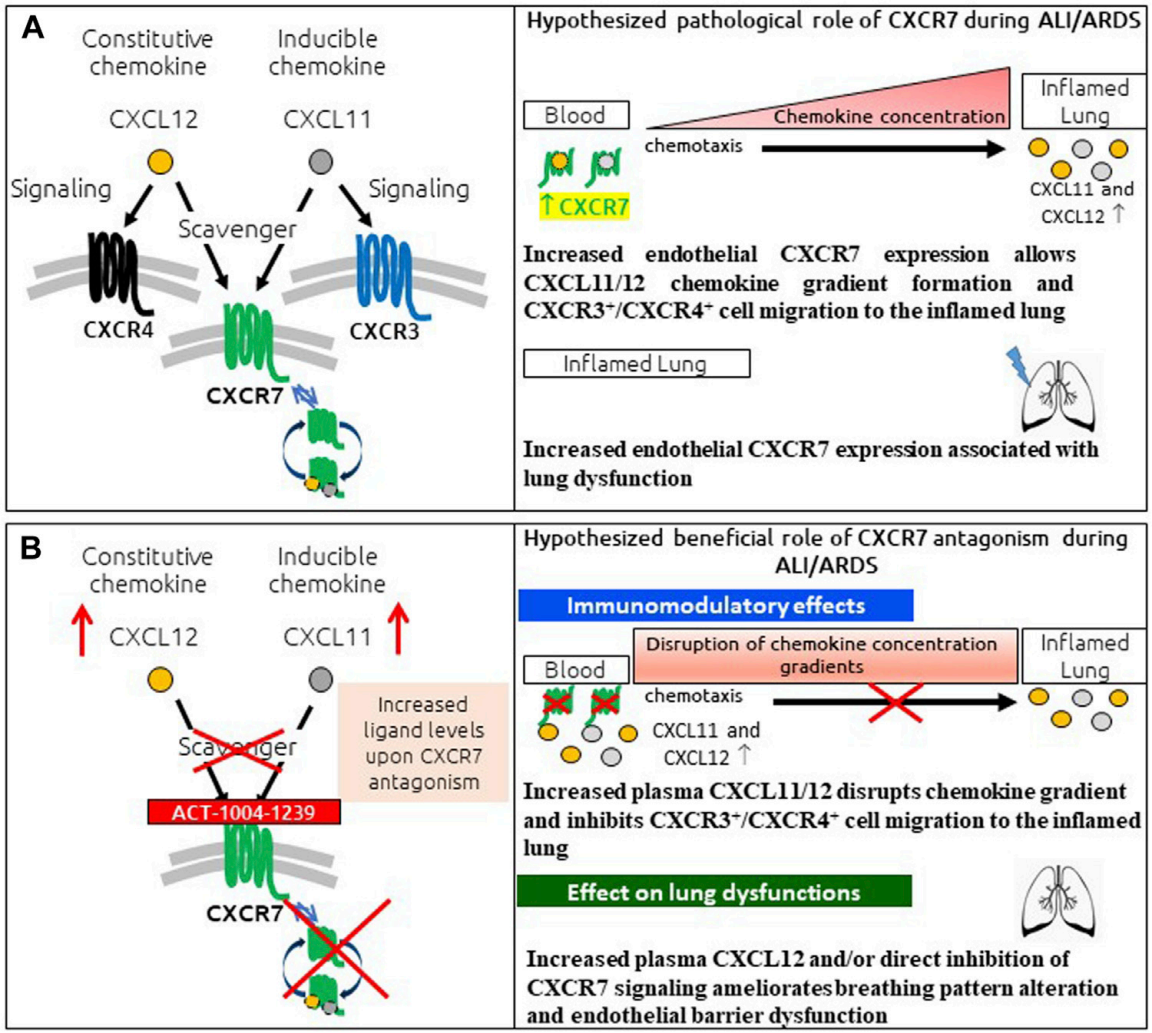
Summary of pathological role of CXCR7 and potential benefit of CXCR7 antagonism during ALI/ARDS. **(A)** CXCR7 functions predominantly as a scavenger receptor for its two ligands: the interferon-inducible chemokine CXCL11 and the constitutive chemokine CXCL12. Binding of its ligands leads to internalization of the CXCR7–ligand complex and ligand degradation. CXCL11 and CXCL12 also bind and activate the signaling chemokine receptors CXCR3 and CXCR4, respectively. The CXCR3/CXCR4/CXCR7 axes play an important role in lung inflammation. CXCR7 scavenging activity tightly regulates the extracellular levels of its ligands, facilitating the establishment and maintenance of CXCL11/12 chemokine concentration gradients and CXCR3^+^/CXCR4^+^ cell migration from the blood to the inflamed lung. In addition, increased CXCR7 expression in the inflamed lung has been reported to be associated with breathing pattern alteration and endothelial barrier dysfunction. **(B)** CXCR7 antagonism with the CXCR7 antagonist ACT-1004-1239 exhibits immunomodulatory effects: by blocking the scavenging activity of the receptor and consequently increasing CXCL11 and CXCL12 plasma concentrations, chemokine gradients are disrupted, inhibiting CXCR3^+^ and CXCR4^+^ cell migration to the inflamed lung. In addition, treatment with ACT-1004-1239, by increasing plasma CXCL12 and/or by direct inhibition of CXCR7 signaling, ameliorates ALI-induced breathing pattern alteration and endothelial barrier dysfunction.

In conclusion, the presented data provide a characterization of the LPS-induced ALI/ARDS model in DBA/1 mice, demonstrating a key role for CXCR7 on pathological hallmarks of human disease (Figure 6A). The scavenging activity of CXCR7 contributed to the establishment and maintenance of CXCL11 and CXCL12 concentration gradients, thereby allowing the recruitment of CXCR3^+^ and CXCR4^+^ leukocytes to the BAL (Figure 6A). Antagonizing CXCR7 with ACT-1004-1239 increased plasma CXCL11 and CXCL12 levels and reduced infiltration of CXCR3^+^ and CXCR4^+^ leukocytes to the BAL (Figure 6B). Furthermore, CXCR7 antagonism reduced vascular permeability and breathing dysfunction (Figure 6B). This broad mechanism of action positions ACT-1004-1239 as a potential new therapy to target main pathological features of human ALI/ARDS.

## Data Availability

The datasets presented in this study can be found in online repositories. The names of the repository/repositories and accession number(s) can be found in the article/Supplementary Material.

## Abbreviations

ALI, acute lung injury; ANOVA, analysis of variance; ARDS, acute respiratory distress syndrome; BAL, bronchoalveolar lavage; bid, twice daily; DCs, dendritic cells; LPS, lipopolysaccharide; NK cells, Natural killer cells; pDCs, plasmacytoid dendritic cells; SEM, standard error of the mean.
