# Supplementary figures and images for: CXCR7 Antagonism Reduces Acute Lung Injury Pathogenesis
Source: Front Pharmacol. 2021 Nov 5;12:748740. doi: 10.3389/fphar.2021.748740 (PMC8602191; doi:10.3389/fphar.2021.748740)

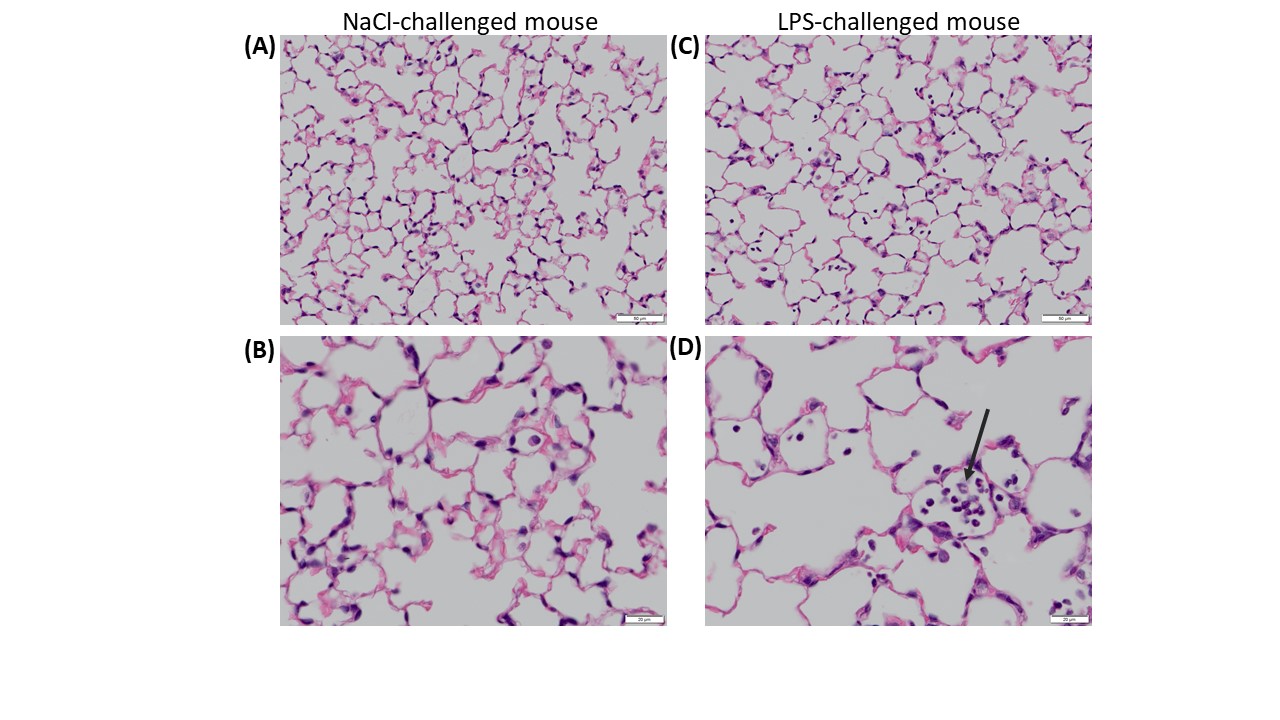

Supplement: Supplementary file 1 [file Image3.JPEG]

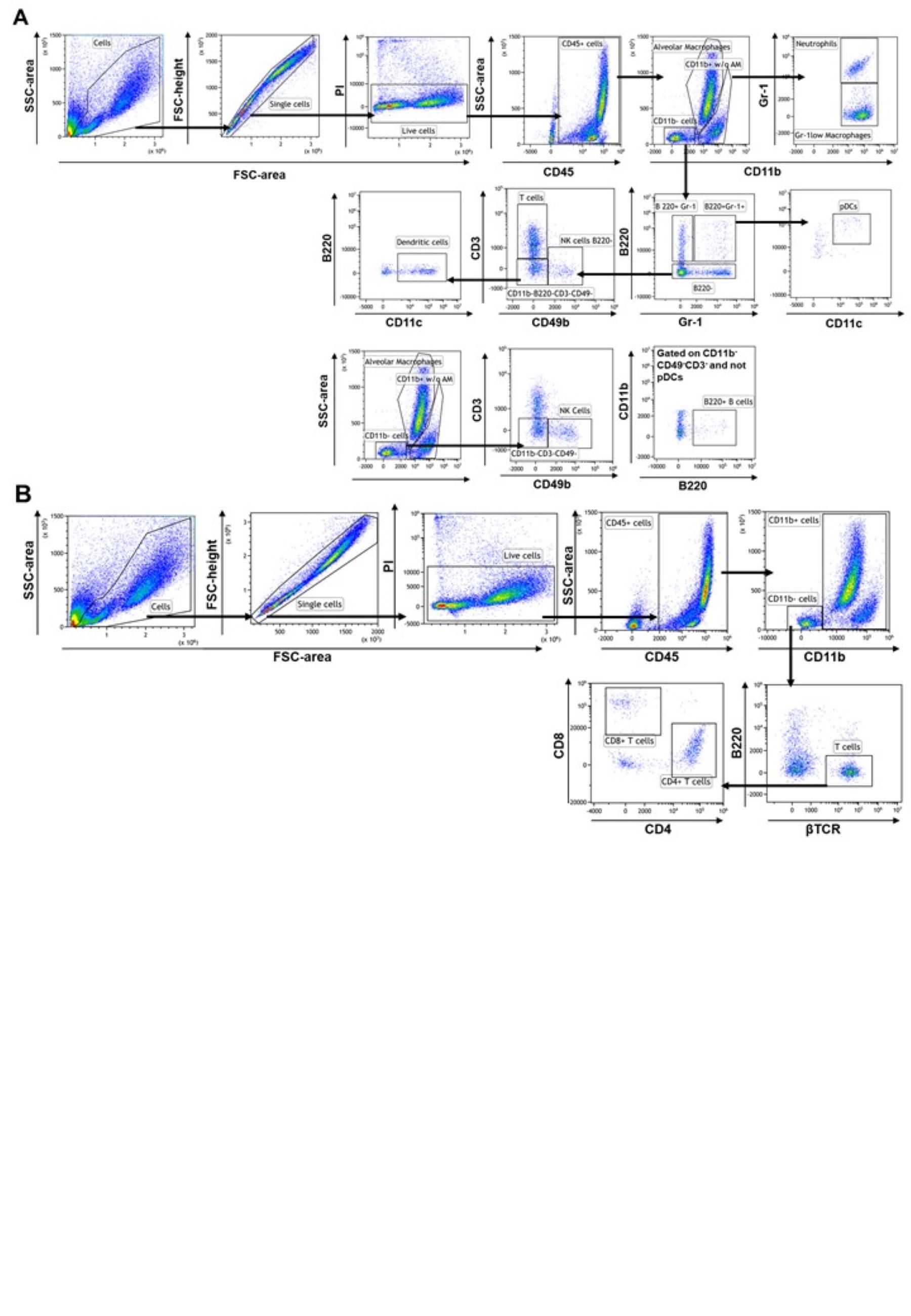

Supplement: Supplementary file 2 [file Image1.JPEG]

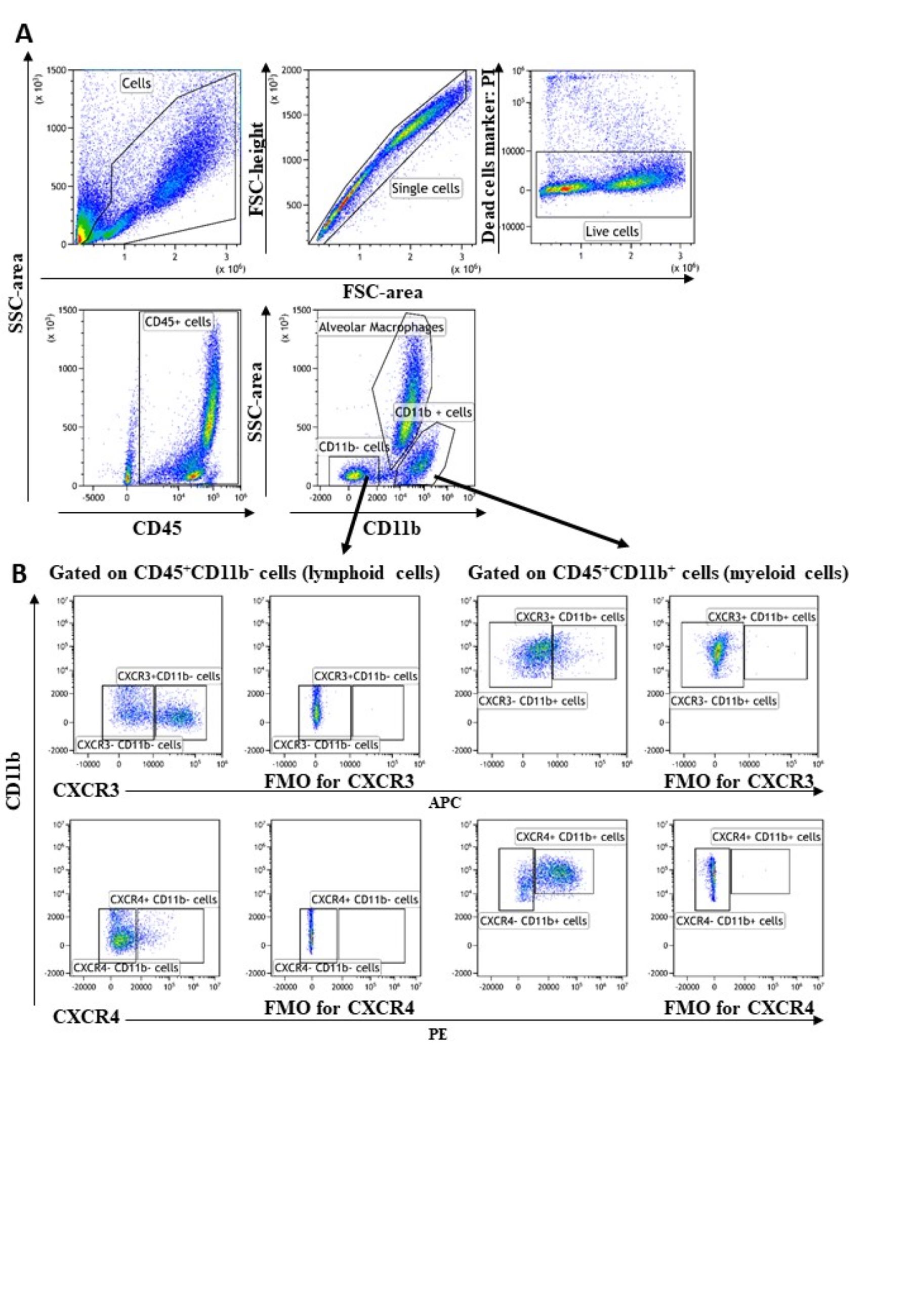

Supplement: Supplementary file 3 [file Image2.JPEG]
