# Supplementary material for: CXCR7 Antagonism Reduces Acute Lung Injury Pathogenesis
Source: Front Pharmacol. 2021 Nov 5;12:748740. doi: 10.3389/fphar.2021.748740 (PMC8602191; doi:10.3389/fphar.2021.748740)
Supplement: Supplementary file 4 [file DataSheet1.DOCX]

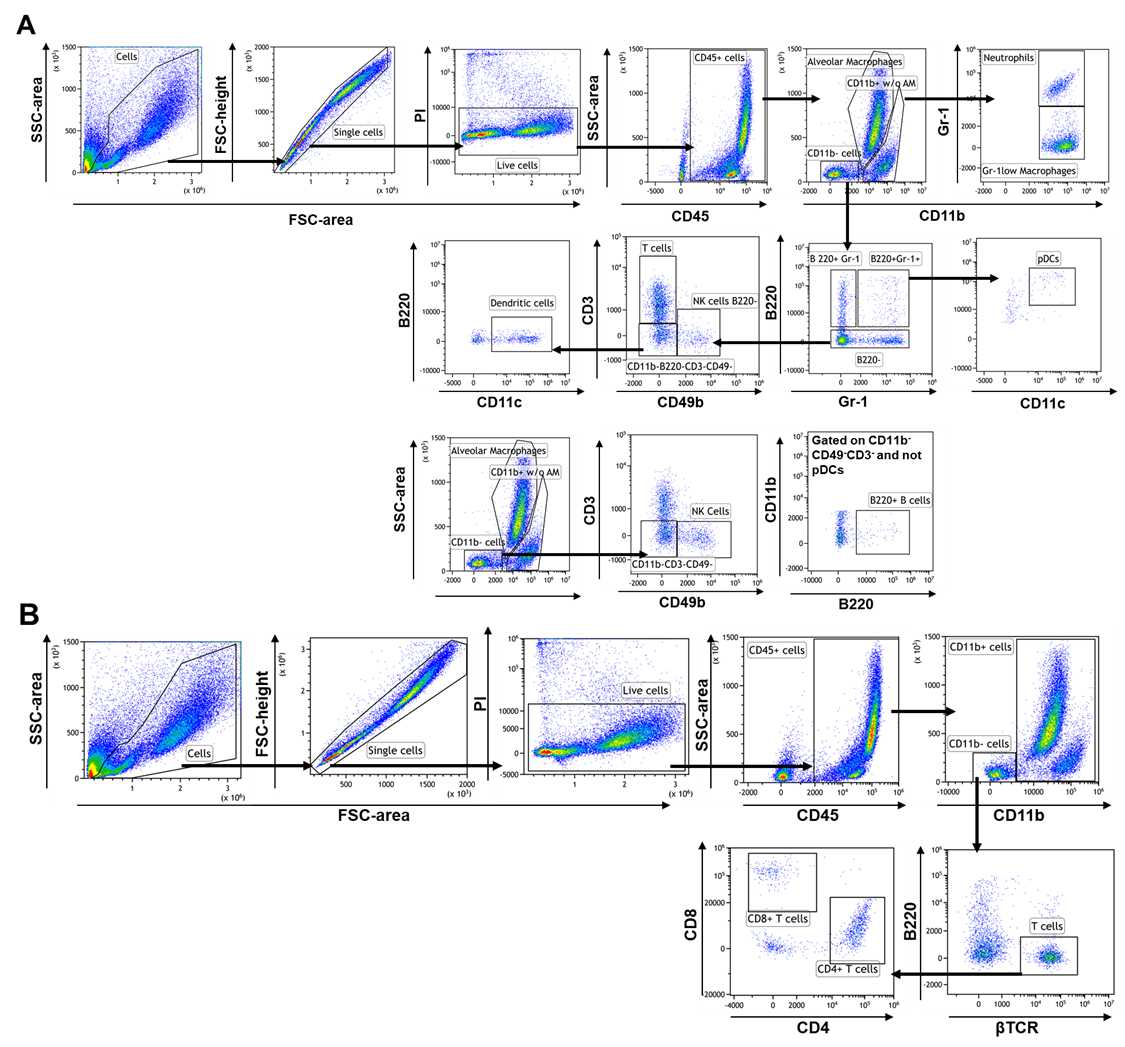


Figure S1. Gating strategy for bronchoalveolar lavage (BAL) cell immunophenotyping. Male DBA/1 mice were exposed to nebulized LPS. BAL samples were collected 5h, 24h, 48h and 72h post LPS challenge and analyzed using flow cytometry. The gating strategy depicts representative plots from vehicle-treated mice, 72h post LPS challenge. (A) After gating on the cell population based on forward scatter (FSC) vs. side scatter (SSC), the exclusion of doublets and dead cells, CD45^+^ viable cells were defined against the SSC. Among CD45^+^ cells, the following populations were gated: alveolar macrophages (CD11b^int^, SSC^high^), macrophages (CD11b^+^, Gr-1^low^), neutrophils (CD11b^+^, Gr-1^high^), plasmacytoid dendritic cells (pDCs) (CD11b^-^, Gr-1^int^, B220^+^, CD11c^+^), T cells (CD11b^-^, B220^-^, CD49b^-^, CD3^+^), classical CD11b^-^ dendritic cells (DCs) (CD11b^-^, B220^-^, CD49b^-^, CD3^-^, CD11c^+^ cells), natural killer (NK) cells (CD11b^-^, CD3^-^, CD49b^+^), and B cells (CD11b^-^, Gr-1^-^, CD49b^-^, CD3^-^, B220^+^). (B) After gating on the cell population based on FSC vs. SSC, the exclusion of doublets and dead cells, CD45^+^ viable cells were defined against the SSC. CD45^+^ cells were then further gated on CD11b vs. SSC, and the CD11b^-^ cells on B220 vs. βTCR to select βTCR^+^B220^-^ T cells.
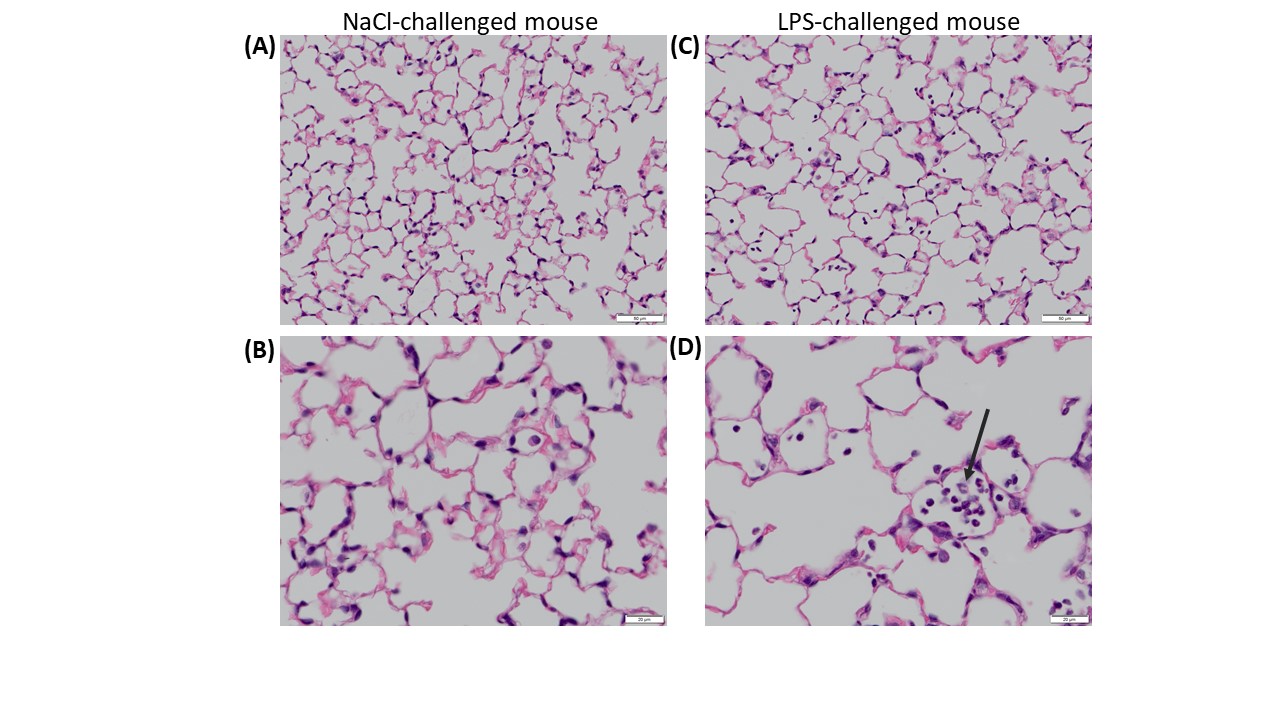
Subpopulation of βTCR^+^B220^-^ T cells were then further defined based on their CD8 vs. CD4 stain.

Figure S2: Representative pictures of lung tissue stained with hematoxylin & Eosin. Male DBA/1 mice were exposed to nebulized NaCL (control) or LPS. 24 hours following challenge, mice were euthanized with pentobarbital and lungs were collected as a whole, filled with 4% paraformaldehyde and then postfixed for 24h, dehydrated with alcohol, and embedded in paraffin wax. Lung paraffin sections of 2 μm were stained with hematoxylin-Eosin and assessed by a pathologist for evidence of inflammation. (A and B) 20x and 40x, respectively from a control mouse challenged with NaCl. (C and D) 20x and 40x, respectively from a mouse challenged with LPS. (scale bar, 20μm in B and D and 50μm inA and C) The arrow shows a neutrophil.


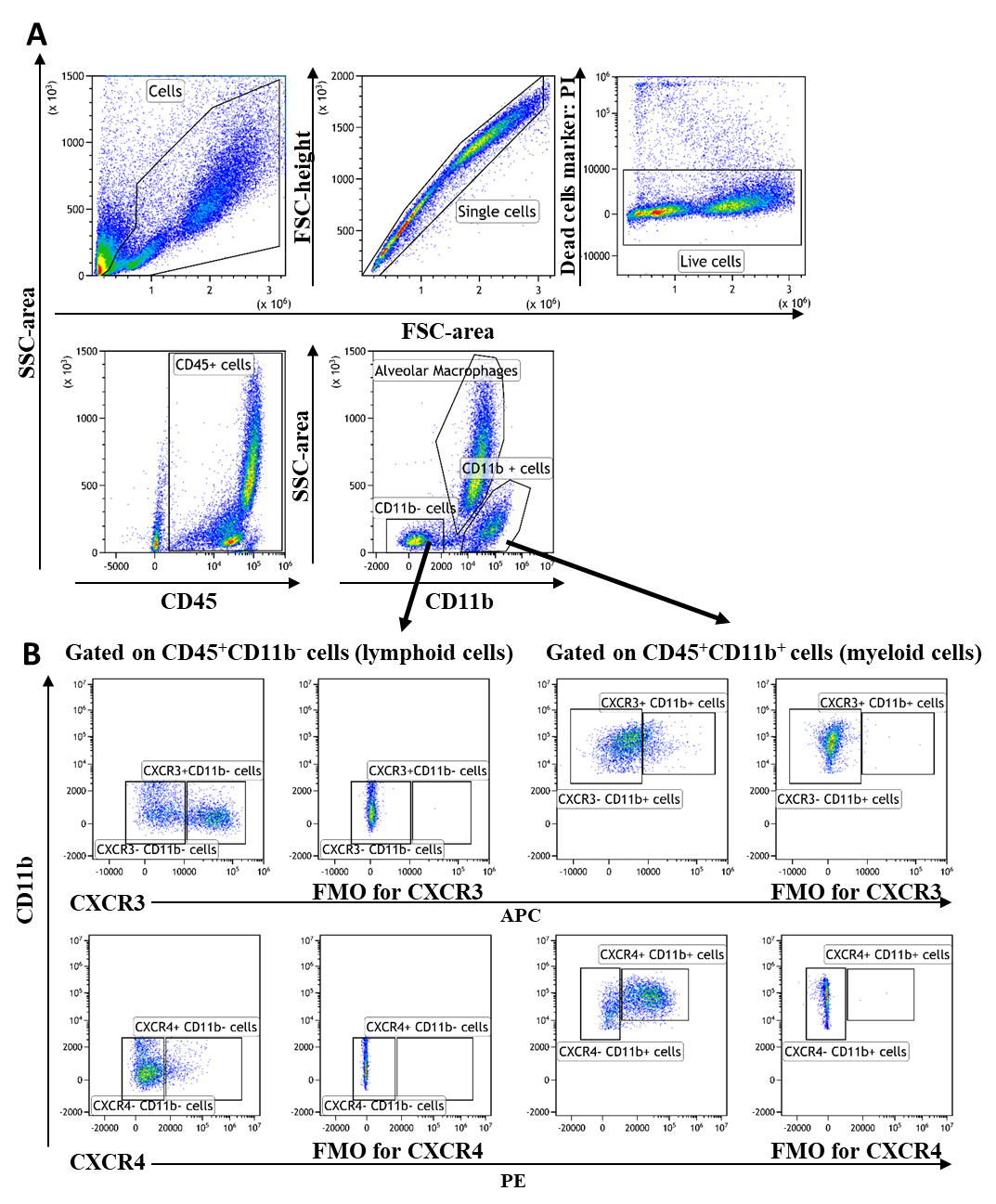


Fig. S3. Gating strategy for bronchoalveolar lavage (BAL) lymphoid and myeloid cell immunophenotyping. Male DBA/1 mice were exposed to nebulized LPS. BAL samples were collected 5h, 24h, 48h and 72h post LPS challenge and analyzed using flow cytometry. The gating strategy depicts representative plots from vehicle-treated mice, 72h post LPS challenge. (A) After gating on the cell population based on FSC vs. SSC, excluding doublets and dead cells, CD45^+^ viable cells were defined against the SSC. Among CD45^+^ cells, the following populations were gated: alveolar macrophages (CD11b^int^, SSC^high^), lymphoid cells (CD11b^-^, SSC^lo^), myeloid cells (CD11b^+^, SSC^lo^).(B) Gating strategy for CXCR3^+^ and CXCR4^+^ BAL lymphoid and myeloid cells based on the FMO control for CXCR3 and CXCR4, respectively.
